# Effects of Different Electroacupuncture Scheduling Regimens on Murine Bone Tumor-Induced Hyperalgesia: Sex Differences and Role of Inflammation

**DOI:** 10.1155/2012/671386

**Published:** 2012-12-20

**Authors:** Branden A. Smeester, Mona Al-Gizawiy, Alvin J. Beitz

**Affiliations:** ^1^Department of Veterinary and Biomedical Sciences, College of Veterinary Medicine, University of Minnesota, St. Paul, MN 55108, USA; ^2^Department of Radiology, Medical College of Wisconsin, Milwaukee, WI 53226, USA; ^3^Department of Veterinary and Biomedical Sciences, University of Minnesota, Room 205 Veterinary Medicine Building, 1971 Commonwealth Avenue, St. Paul, MN 55108, USA

## Abstract

Previous studies have shown that electroacupuncture (EA) is able to reduce hyperalgesia in rodent models of persistent pain, but very little is known about the analgesic effects and potential sex differences of different EA treatment regimens. In the present study, we examined the effects of five different EA treatments on tumor-induced hyperalgesia in male and female mice. EA applied to the ST-36 acupoint either twice weekly (EA-2X/3) beginning on postimplantation day (PID) 3 or prophylactically three times prior to implantation produced the most robust and longest lasting antinociceptive effects. EA treatment given once per week beginning at PID 7 only produced an antinociceptive effect in female animals. The analgesic effect of EA-2X/3 began earlier in males, but lasted longer in females indicating sex differences in EA. We further demonstrate that EA-2X/3 elicits a marked decrease in tumor-associated inflammation as evidenced by a significant reduction in tumor-associated neutrophils at PID 7. Moreover, EA-2X/3 produced a significant reduction in tumor-associated PGE_2_ as measured in microperfusate samples. Collectively, these data provide evidence that EA-2X/3 treatment reduces tumor-induced hyperalgesia, which is associated with a decrease in tumor-associated inflammation and PGE_2_ concentration at the tumor site suggesting possible mechanisms by which EA reduces tumor nociception.

## 1. Introduction

Although acupuncture remains a controversial subject in Western Medicine, it is widely used in today's clinical practice [1] and appears to be a viable option for the treatment of many types of pain [2–4] including cancer pain [5, 6] and chemotherapy-induced peripheral neuropathy [7, 8]. Cancer is a highly destructive and infiltrative disease resulting in uncontrolled proliferation, apoptotic deregulation, and invasive metastasis. Up to 90% of patients with metastatic or advanced stage cancer will experience significant cancer-related pain. Bone metastases are often associated with dramatic reductions in quality of life, mobility, and independence as well as excruciating refractory pain [9]. In a recent pan-European survey, 63% of patients on prescribed analgesics experienced breakthrough cancer pain [10]. While some progress had been made in attempting to elucidate the mechanisms responsible for tumor-induced bone pain [11–13], the potential role of inflammation in bone tumor pain and the mechanisms underlying breakthrough pain are poorly understood. Furthermore, although opiates are a mainstay of cancer pain treatment, recent work has shown that morphine accelerates sarcoma-induced bone pain, bone loss, and spontaneous fractures [14]. In this regard, it is critical that further research be done to better understand bone cancer mechanisms, how inflammation affects bone cancer progression, and if alternative treatments can be found that both relieve pain and reduce tumor burden. 

While acupuncture has been around for thousands of years, we are only now starting to get a glimpse of the mechanisms underlying its effects [15] particularly those associated with pain relief [16, 17]. As we begin to study the mechanisms underlying acupuncture's analgesic effects, there are important questions that arise regarding whether different acupuncture treatment regimens have different effects on pain and whether sex differences exist related to these treatment regimens. In the present study we hypothesized that different treatment regimens would have differential antinociceptive effects on tumor-induced pain. We also wanted to determine if there were sex differences among different acupuncture treatment schedules, since this is an area that has received little attention in the literature. Thus, the first portion of the present study was designed to examine the effectiveness of five different EA treatment regimens in reducing bone tumor pain and to also determine whether these regimens had differential effects in male versus female animals. 

Acupuncture also appears to affect the immune system and therefore can have a direct effect on inflammation [18, 19], including reducing neutrophil invasion during sepsis [20]. The role of inflammation in carcinogenesis has been extensively investigated and well documented [21–23]. Neurogenic inflammation appears to underlie certain forms of colon cancer [24]. In this regard, the inflammatory state is necessary to maintain and promote cancer progression and to accomplish the full malignant phenotype, including tumor tissue remodeling, angiogenesis, metastasis, and the suppression of the innate anticancer immune response [25]. While several studies now support acupuncture's ability to reduce inflammation [26, 27], its potential effect on the inflammatory stage of cancer or the inflammatory component of cancer pain has not been examined. In the present study, we hypothesized that low-frequency electroacupuncture (EA) has an anti-inflammatory effect on neutrophil and macrophage recruitment during early tumor development and that acupuncture-induced inhibition of tumor-associated inflammation reduces tumor-induced hyperalgesia. To test these hypotheses, we utilized an established hind paw osteosarcoma mouse model developed at the University of Minnesota [28, 29] in the second part of this study to examine the above relationships *in vivo*. 

## 2. Materials and Methods

### 2.1. Cells

The K7 M2 cell line was a kind gift from Dr. Khanna [30] and was derived from the K7 cell lines initially established from a spontaneous murine OGS [31]. National Collection of Type Cultures (NCTC) clone 2472 fibrosarcoma cells, originally derived from a connective tissue tumor in a C3H mouse, were obtained from the American Type cell Culture Collection (Rockville, MD). Each cell type is syngeneic to its particular mouse line. Cells were grown and maintained in accordance with standard cell culturing techniques. K7 M2 cells were grown to 80%–90% confluence in 75 cm^2^ flasks (Corning, Lowell, MA) in Dulbecco's Modification of Eagles Medium (Invitrogen, Carlsbad, CA) containing 1% penicillin/streptomycin and 10% fetal bovine serum prior to implantation. NCTC 2472 cells were grown using a similar protocol, fortified with 10% horse serum, sodium bicarbonate, and also grown to 80%–90% confluency. Cell cultures were housed in a water-jacketed incubator with 5% carbon dioxide at 37°C.

### 2.2. Animals

The present study used a well-established mouse hind paw model of bone cancer pain [13, 28] to examine the effects of EA on tumor-induced nociception. A total of 104 female and 166 male young adult BALB/c and 20 male C3H mice (National Cancer Institute, Bethesda, MD) were used in this study. The number of animals in each group that were used for each experiment is summarized in Table 1.

The inbred BALB/c mouse strain was used in these experiments because this mouse strain is syngeneic to the K7 M2 osteosarcoma used in these experiments and thus allows these cells to grow tumors without rejection. Osteosarcoma tumors were studied in the present investigation because osteosarcoma is an extremely painful bone tumor that affects children and teenagers and an equally painful tumor in dogs, and we wanted to investigate the potential for EA to treat osteosarcoma-induced bone pain; C3H/He mice were used for the microperfusion experiments in the present study because they are syngeneic to the fibrosarcoma cells used for these experiments. Thus, BALB/c mice were used for behavioral and immunohistochemical experiments, while C3H mice were used for microperfusion experiments. The C3H mice were selected for the microperfusion experiments, because based on preliminary data we are able to obtain more robust and reproducible results when performing microperfusion experiments on fibrosarcoma tumors as compared to osteosarcoma tumors *in vivo*. Animals were maintained on a 12 hr light/dark cycle with food and water *ad libitum. *Procedures were performed in accordance with the guidelines recommended by the International Association for the Study of Pain, and all experimental protocols were approved by the Animal Care and Use Committee at the University of Minnesota.

### 2.3. Implantation

Cells were prepared for implantation by first pouring off the culture medium and washing with PBS. Trypsin was added to flask and placed in incubator for 3 min. Upon detachment, cells were suspended in an ample amount of culture medium to terminate enzymatic activity and centrifuged for 10 min at 1,000 g. Osteosarcoma and fibrosarcoma cells were resuspended in a known amount of PBS for counting, quantified on a hemocytometer, pelleted and re-suspended in PBS. Osteosarcoma cells were resuspended to a final concentration of 1 × 10^6^ cells in 50 *μ*L, while fibrosarcoma cells were re-suspended to a final concentration of 2 × 10^5^ cells in 10 *μ*L. Initially, animals were anesthetized in a plexi-glass chamber using 3% isoflurane in 3 L/min oxygen. Once each mouse was completely anesthetized, a maintenance rate of 2% isoflurane in 1.5 L/min oxygen was maintained during the short implantation procedure. Tumor cells were manually injected by boring into the calcaneus bone using a 29.5 gauge needle connected to a sterile 0.3 mL insulin syringe as previously described [28]. A control group of BALB/c mice (*n* = 10) received an injection of saline into the calcaneus bone rather than tumor cells. These mice were used for the initial behavioral testing. Following injection, mice were allowed to recover in cages on a heating pad. Animals showing any signs of dysfunction (e.g., problems with ambulation, lethargy, or excessive bleeding) or any animals in which the tumor did not grow were euthanized and removed from the study. This occurred in less than 5% of the animals used in this study.

### 2.4. Behavioral Testing

Animals were placed under clear glass cups on a wire grid and allowed to acclimate for 30 min. Mechanical hypersensitivity was tested using a von Frey filament # 2.83, which produces a force of 62–95 mg [32], and was applied to the plantar surface of each hind paw with enough force to cause it to bow slightly. Starting with the right hind paw, the numbers of positive responses out of a total of 10 applications were recorded. Baseline von Frey measurements were obtained prior to tumor implantation or saline injection into the calcaneus. Subsequent von Frey measurements were taken 30 min after each electroacupuncture treatment as well as on days 3, 7, 10, 14, 17, and 21. Animals with saline injection into the calcaneus did not receive any EA treatment, but rather served as controls for tumor cell implantation and tumor-induced nociception. Behavioral assessments were conducted during the light cycle at approximately the same time each day. The investigator performing the von Frey testing was blinded to the animal and EA treatment (Tumor-EA versus Tumor-Sham EA versus Tumor-non-acupoint EA versus No Tumor-No EA, i.e., saline implantation).

### 2.5. Transcardiac Perfusions

Mice were deeply anesthetized with 50 mg/kg sodium pentobarbital (Nembutal; Ovation Pharmaceuticals, Inc., Deerfield, IL) injected intraperitoneally. When mice were no longer responsive to paw pinch, the thoracic cavity was quickly accessed via the abdomen to isolate the heart. A 21-gauge butterfly catheter (Terumo Medical Corporation, Somerset, NJ) was inserted into the left ventricle and secured using forceps. The right atrium was punctured to allow both drainage of blood and fixative. Fifteen mL of ice cold PBS was perfused followed by 30 mL of 10% neutral-buffered formalin at a rate of 3 mL/min. Following perfusion, tumors were excised, postfixed in the same fixative overnight at 4°C, and cryoprotected in 30% sucrose for 24–48 hours at 4°C prior to tissue sectioning. 

### 2.6. Microperfusions

Microperfusion of the tumor was performed as previously described [28] in order to examine the potential effect of EA on the concentration of prostaglandin E_2_ in the extracellular fluid of a hind paw fibrosarcoma. Previous microperfusion studies in our lab have been conducted on C3H mice, and, thus, we have established baseline levels for prostaglandins, cytokines, and other potential pain mediators in this mouse line. Moreover, we have found in preliminary experiments that the microperfusion technique has a higher success rate in C3H mice with fibrosarcoma tumors then in BALB/c mice with osteosarcomas. Since hind paw fibrosarcoma tumors in C3H mice produce a level of mechanical hyperalgesia that is similar to that of osteosarcoma tumors [28] and since EA also produces antinociception in this mouse strain, we evaluated the effect of EA on the concentration of prostaglandin E_2_ in fibrosarcoma tumors rather than osteosarcomas. Briefly, we implanted a perfusion microprobe consisting of a 29.5-gauge stainless steel hypodermic needle with a 2 mm opening in the middle, extending a “push-pull” microperfusion design as previously described [28] into the tumor site, and secured the probe with super glue. Using two peristaltic pumps (Rabbit Plus, Rainin Instrument Co, Columbus, OH), we utilized this push-pull technique to collect tumor secretions at various time points following EA or sham treatments. The tumor was perfused with a modified Ringer's Solution at a flow rate of 10 *μ*l/min. Preliminary experiments have determined that this setting on the outlet pump maintained a constant pressure at the microprobe opening and prevented clogging of the probe. Heparin (2 *μ*l) was used to prevent potential blood clotting in the microprobe. Polyethylene tubing (PE-10) was used to connect the peristaltic pumps with the microprobe. Animals were initially anesthetized in a plexi-glass chamber using 3% isoflurane in 3 L/min oxygen. Once animals were anesthetized as measured by paw pinch, a maintenance rate of 2% isoflurane in 1.5 L/min oxygen was used during the microperfusion procedure. After a 30 min equilibration period, fractions were collected in 30-minute increments before treatment (BL) and at two time points following EA or sham treatment. Samples were collected and augmented with a protease inhibitor mixture (Sigma, St. Louis, MO). They were then stored at −80°C for later analysis by ELISA assay.

### 2.7. EA Stimulation Parameters

 For EA, sham, or nonacupoint treatment, animals were initially anesthetized with 3% isoflurane/3 L oxygen and then maintained at 2% isoflurane/1.5 L oxygen for the length of the EA, sham, or nonacupoint procedure (30 min). For the EA and sham-EA acupuncture groups, two stainless steel intradermal needles (SEIRIN-America, Weymouth, MA) were inserted to a depth of 3 mm into the hind limb at the ST-36 Zusanli acupoint located between the tibia and fibula, approximately 5 mm lateral to the anterior tubercle of the tibia. Rather than testing multiple acupoints and examining their effects on tumor nociception, we focused on one point, Zusanli, for the purposes of this study. This allowed us to evaluate differences in the frequency and timing of the EA application, rather than the effect of stimulating different individual acupoints or simultaneous stimulation of multiple acupoints. The Zusanli acupuncture point was selected because it has been used to evaluate the effect of EA on a variety of pain conditions in rodent models of persistent and chronic pain and has been found to produce significant analgesia [33, 34]. In the EA groups, the Zusanli acupoint was stimulated using a Maxtens 1000 dual channel stimulator with a 4 Hz pulse rate, 100 *μ*s pulse width, for a total of 30 min. A similar paradigm was used to stimulate a nonacupoint in Group four (see the following).

#### 2.7.1. Electroacupuncture Treatment Regimens

To our knowledge, there are very few controlled studies in the literature that have examined the effects of different acupuncture regimens (treatment parameters) on pain. The present study examines and compares the potential anti-nociceptive effects of five different EA treatment paradigms administered at the ST-36 acupoint in a rodent model of bone cancer pain. Three of our regimens, EA given twice per week and EA given once per week, are based on those used clinically to treat pain [5, 35]. The five treatment protocols were as follows: EA administered once weekly starting at PID 7 (EA-1X/7); EA administered twice weekly starting at PID 3 (EA-2X/3) or PID 5 (EA-2X/5); EA administered once on day 1 following tumor cell implantation (EA Once/1); and EA administered 3 times prior to tumor cell implantation with no treatment following implantation, which we designated as the prophylactic (EA Pro) treatment subgroup. Male and female osteosarcoma tumor-bearing animals were divided into the following four experimental and control groups: *Group 1:* an EA treated tumor group, which consisted of 5 subgroups of animals all implanted with tumor cells but each receiving a different EA treatment regimen as described previously (EA-1X/7, EA-2X/3, EA-2X/5, EA Once/1, or EA Pro); *Group 2:* a sham-EA treated tumor group, which consisted of 5 subgroups of animals all implanted with tumor cells, but each receiving a different sham-EA treatment regimen (similar to the EA treatment regimens described previously) that involved implanting a acupuncture needle into ST-36, but not applying any electrical current (Sham-1X, Sham-2X/3, Sham-2X/5, Sham Once/1, and Sham Pro); *Group 3*: a Tumor-No EA treatment group (mice that were implanted with tumor cells, but received no EA or sham treatment and were subsequently euthanized at day 21 after implantation; *n* = 10); and *Group 4:* a nonacupoint EA treated tumor group (N/A-2X/3; *n* = 10), which received EA twice a week in a nonacupoint located on the base of the tail as previously described by Kim et al. [36]. Similar to group one, the non-acupoint treatment group received twice weekly EA at the non-acupoint site for a period of 21 days. 

Sham treatment consisted of acupuncture needles being inserted into the ST-36 acupoint for 30 min, without application of electrical stimulation. Previous studies indicate no significant differences in nonpenetrating sham needles, hence our omission of this control. [37–39]. However, in accordance with the reasoning that acupuncture analgesia is dependent on the mechanical signal elicited by needle manipulation, our sham EA group had the needles inserted, but there was no EA current or mechanical stimulation [40–44].

### 2.8. Immunohistochemistry for Immunocytes

Because inflammation is a component of many tumor types and since acupuncture has been shown to reduce inflammation, we hypothesized that acupuncture may reduce tumor-induced nociception by reducing tumor-associated inflammation. Here we use immunocytochemistry to analyze the potential effects of EA on neutrophils and macrophages at the tumor site. Since the EA-2X/3 treatment schedule provided the most effective and long lasting antinociceptive effect, this treatment schedule was selected to examine the potential effects of EA on tumor-associated inflammation. Osteosarcoma tumor-bearing hind limbs were removed immediately after transcardiac perfusion, postfixed in 10% neutral-buffered formalin overnight at 4°C, and then placed in 30% sucrose for cryoprotection. Consecutive tissue sections (40 microns) through the distal third (2.5 mm), the middle third (2.5 mm), and the proximal third (2.5 mm) of the tumor were cut transversely using a cryostat. Tissue sections through each third of the tumor were separated by 300 *μ*m increments to avoid double counting of cell numbers. This allowed us to sample the entire tumor (average tumor diameter was 7.5 mm in diameter). Thus, a total of 30 sections (10 from the distal third, 10 from the middle third, and 10 from the proximal third) through the tumor of each mouse were collected and processed using a standard immunofluorescent procedure. Six tumor sections were randomly selected from each third of the tumor and were blocked for 1 hr with 0.3% Triton X-100/PBS (TPBS) and 5% donkey serum at RT. Three of the randomly selected tissue sections from each third of the tumor (9 sections total) were then incubated overnight at 4°C with rat anti-mouse NIMP-R14 (1 : 50; Abcam, Cambridge, MA) to specifically label neutrophils. The remaining nine randomly selected tissue sections from the tumor were incubated overnight with rat anti-mouse MOMA-2 (1 : 50; Abcam, Cambridge, MA) to specifically label macrophages/monocytes at the tumor site. Following 3 × 10 min PBS washes, sections were incubated for 2 hr at RT using Cy 3-conjugated AffiniPure Donkey Anti-Rat IgG (1 : 400; Jackson Immuno, West Grove, PA). Sections were washed 3 × 10 min in PBS, air-dried for 15 min, and mounted using Pro-Long Gold mounting medium containing 4′,6-diamidino-2-phenylindole (Invitrogen, Carlsbad, CA). In addition, 2 tissue sections from each third of the tumor were used as controls and were processed using immunocytochemistry with either the primary or secondary antibody left out of the process to determine possible nonspecific staining.

### 2.9. Immunocyte Quantitative Analysis

The quantification of neutrophils and macrophages in tumor sections was performed based on the computer-assisted cell counting method recently developed by Väyrynen and coworkers [45]. Peripheral osteosarcoma tumor sections from the left hind paw were analyzed at 40X magnification using a Nikon Eclipse 80i (Nikon, Melville, NY) microscope to determine the densities of neutrophils and macrophages. Tissue sections were thresholded using preset software parameters in order to insure consistency among fields of view and slides per animal. Three random fields were selected from the peripheral portion of the tumor in each section, and the density of cells in each field was determined. A total of 7 sections per antibody were analyzed per animal. In addition, 6 control sections were analyzed from each animal in which the primary or secondary antibody was omitted from the immunohistochemical processing. For these controls, the tissue was incubated in blocking solution without the primary antibody or with PBS rather than the secondary antibody. These sections served as controls for nonspecific staining. Since we did not have access to the original antigens used to generate the antibodies used in this study, we were unable to perform appropriate adsorption controls. Nikon ACT-1 (Nikon, Melville, NY) software was used to acquire the images for processing. Individual cell types (neutrophils or macrophages) were identified by a brighter immunofluorescent staining pattern visible against the darker background. In addition, we used a DAPI counterstain and H&E staining to positively identify cell types. Neutrophils were characterized by their multilobed nucleus and pink cytoplasm, while macrophages are larger cells with pleomorphic nuclei and pale gray-blue cytoplasm that often contain vacuoles. ImageJ software (National Institutes of Health, Bethesda, MA) was utilized for pixel density collection and analysis. The quantitative analysis that was performed on tumor sections was done entirely by an investigator blinded to the experimental conditions.

### 2.10. Enzyme-Linked Immunosorbent Assay (ELISA) Measurement of Prostaglandin E_2_ Supernatant

 A serological testfor prostaglandin E_2_ was performed using a commercially available EIA kit (Caymen Chemical Company, Ann Arbor, MI). An anti-E_2_ monoclonal antibody was used on microperfusate samples collected from the hind paw fibrosarcoma tumor 30 minutes prior to the EA procedure (to determine baseline concentrations) as well as three consecutive 30-minute fractions during and after treatment. Samples were run in duplicate, read at 540 nm on a photospectrometer, and analyzed using Caymen Chemical company software. This analysis was performed by an investigator blinded to the experimental condition.

### 2.11. Statistical Analysis

Complete statistical analyses of all data sets were carried out. Comparisons between groups were performed using a two-way ANOVA with post hoc comparisons using Bonferroni's method. For single time point comparisons between groups or within a group, an unpaired Student's *t*-test was employed. Wherever necessary, a paired Student's *t*-test was used to test for statistical significance at individual time points. Data was analyzed and graphed using Prism 5.0 (GraphPad Software, La Jolla, CA). The level of significance was set at *P* ≤ 0.05.

## 3. Results

### 3.1. Tumor-Induced Hyperalgesic Behavior

**Figure 1:**
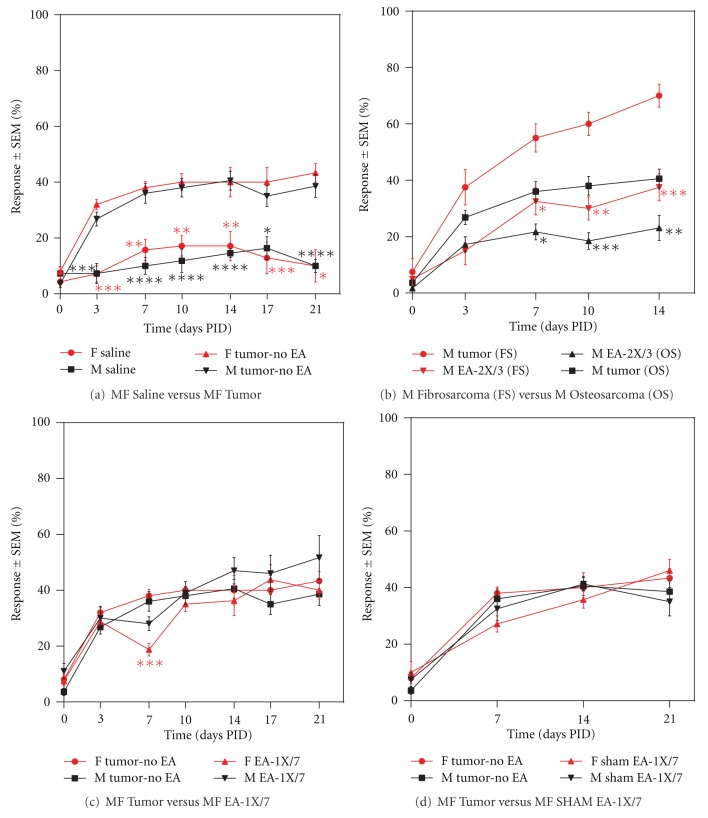
Graphs illustrating (a) tumor-induced mechanical hyperalgesia from postimplantation day (PID) 3 to 21 in male and female mice as measured using a von Frey filament applied 10 times to the hind paw. Pronounced hyperalgesia is evident as early as PID 3 and extends through PID 21 in both male and female animals (*n* = 15/group) as compared to their saline controls (*n* = 5/group, *P < 0.05, **P < 0.01, ***P < 0.001, and ****P < 0.0001); (b) a comparison of the effect of electroacupuncture treatment given twice per week beginning on PID 3 (EA-2X/3) on tumor-induced mechanical hyperalgesia in Bulb/c osteosarcoma male mice (*n* = 18/group) compared to C3H fibrosarcoma male mice (*n* = 4/group). Electroacupuncture produced a significant decrease in tumor-induced hyperalgesia in both mouse strains (*P < 0.05, **P < 0.01, ***P < 0.001). Note that Bulb/c mice were used for the behavioral and immunohistochemical experiments, while C3H mice were used for the microperfusion experiments in this study; (c) the effect of EA administered once per week starting on PID 7 on tumor-induced hyperalgesia; and (d) the effect of sham acupuncture administered once per week starting at day 7 on tumor-induced hyperalgesia. EA-1X/7, but not sham EA, produces an anti-nociceptive effect only in female animals and only on PID 7. Data is presented as the mean % response ± SEM.

Implantation of osteosarcoma cells into the hind paw of BALB/c mice induced hyperalgesia to normally nonnoxious mechanical stimuli as measured using a von Frey monofilament (2.83) when compared to their saline injected controls (Figure 1(a)); *P < 0.05, **P < 0.01, ***P < 0.001, ****P < 0.0001; C3H fibrosarcoma-induced hyperalgesia data is shown in Figure 1(b) and is similar to that published previously [28]. This tumor-induced mechanical hyperalgesia, as defined by a significant increase in the number of responses to von Frey monofilament application, was evident as early as postimplantation day 3 (PID) and continued throughout the duration of the experiment. 

### 3.2. Osteosarcoma versus Fibrosarcoma Hyperalgesia Control

Implantation of either the K7M2 cell line or the NCTC 2472 cell line produced significant tumor-induced mechanical hyperalgesia through PID 14 (Figure 1(b)). There was no significant difference in the amount of hyperalgesia between the two cell lines (Figure 1(b); *P* > 0.05). EA-2X/3 produced a significant decrease in tumor-induced hyperalgesia in both fibrosarcoma and osteosarcoma implanted mice.

### 3.3. Effect of Different Treatment Regimens: EA Treatment at ST-36 Significantly Attenuates Tumor-Induced Hyperalgesia 

#### 3.3.1. EA-1X/7 (EA Administered Once per Week Beginning at PID 7)

EA-1X/7 produced a significant decrease in tumor-induced mechanical hyperalgesia in both male and female animals that was evident at 15 min after EA stimulation, peaked at 30 min post-treatment and returned to pre-EA levels by 60 min after treatment (data not shown). This immediate antinociceptive effect of EA was also evident in the EA-2X/3, EA-2X/5, and EA-once/1 treated animals, but not in any of the sham-EA treated animals. However, when the effects of treatment over time were evaluated, EA-1X/7 (Figure 1(b)) significantly attenuated mechanical hyperalgesia only on PID 7 in female cancer-bearing animals when compared to the Tumor-No EA control group (**P < 0.01). Conversely EA treatment given once a week had no effect on tumor-induced hyperalgesia in male tumor mice at any of the time points tested. Sham treatment given once per week beginning on day 7 had no effect on tumor-induced mechanical hyperalgesia in male or female animals (Figure 1(c)).

#### 3.3.2. EA-2X/3 (Electroacupuncture Administered Twice per Week Beginning PID 3)

**Figure 2:**
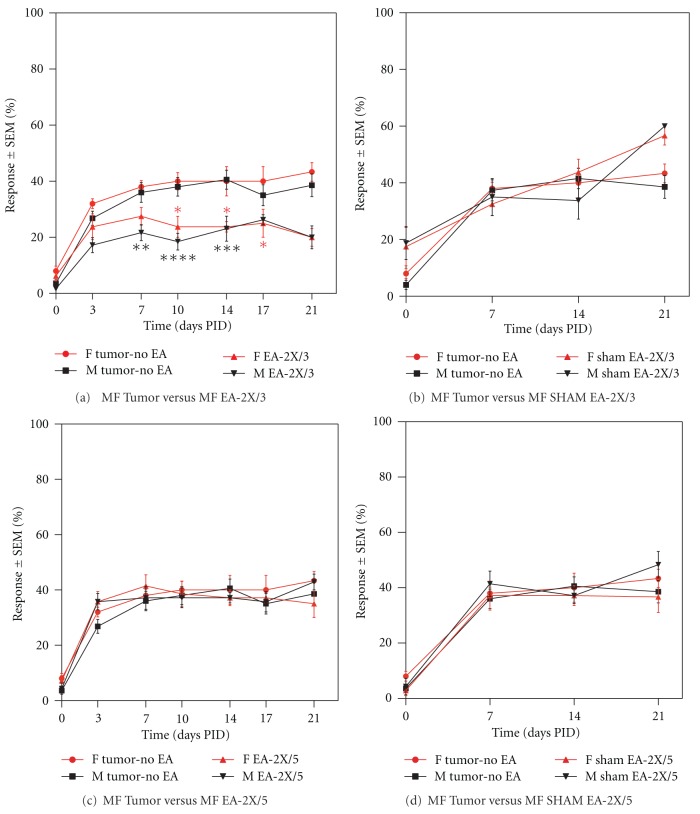
Graphs illustrating the effect of electroacupuncture (a), (c) or sham EA (b), (d) given twice per week beginning on PID 3 (EA-2X/3) or PID 5 (EA-2X/5) on tumor-induced hyperalgesia. EA-2X/3, but not EA-2X/5 had an early and more robust effect on male (M) animals, but had a longer lasting effect on female (F) animals ((a); *n* = 12–18/group). EA-2X/3 produced the longest lasting anti-hyperalgesic effect of all the regimens tested. Neither sham EA-2X/3 nor sham EA-2X/5 had any effect on tumor-induced nociception. Data is presented as mean % response ± SEM, *P < 0.05, **P < 0.01, ***P < 0.001, and ****P < 0.0001 as compared to the tumor-no EA control group.

In male mice, a significant reduction in tumor-induced hyperalgesia was observed beginning at PID 7 as compared to the Tumor-No EA control group, and this reduction was evident through PID 14. In female tumor mice, a significant reduction in tumor-induced hyperalgesia was first observed at PID 10, and this continued through PID 17 (Figure 2(a); *P < 0.05, **P < 0.01, and ****P < 0.0001). Overall, male animals were more sensitive to EA-2X when compared to female counterparts, and, thus, this regimen produced an earlier and more robust antihyperalgesic effect in males. EA-2X was the only treatment regimen that produced significant analgesia at the 14 PID time point in both male and female animals, and this analgesic effect remained through PID 17 in female mice. In contrast, sham EA treatment given twice per week beginning at day 3 had no effect on tumor-induced mechanical hyperalgesia (Figure 2(b)).

#### 3.3.3. EA-2X/5 (Electroacupuncture Administered Twice per Week Beginning PID 5)

 Unlike the effect of EA-2X/3 treatment, EA given twice per week beginning on PID 5 produced no long-term analgesic effect in either male or female animals (Figure 2(c)). Similarly, sham EA-2X/5 had no effect on tumor-induced mechanical hyperalgesia (Figure 2(d)).

#### 3.3.4. EA-ONCE/1 (Electroacupuncture Administered Once at PID 1)

**Figure 3:**
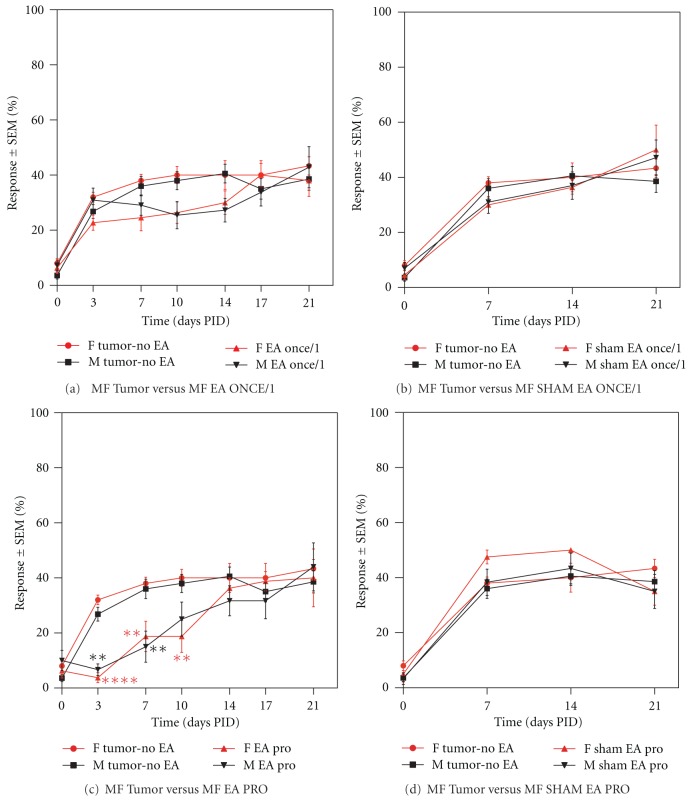
Graphs illustrating the effect of EA and Sham EA administered once on PID 1 ((a) and (b), resp.) and EA or sham EA administered prophylactically prior to tumor cell implantation ((c), (d), resp.) on tumor-induced mechanical hyperalgesia. Sham electroacupuncture produced no significant effects on mechanical hyperalgesia when compared to tumor controls, *n* = 6–12/group. Neither EA once/1 (a) nor Sham EA Once/1 (b) had any significant effect on tumor nociception. EA Pro produced an early anti-nociceptive effect beginning on PID 3 in both male and female animals (c). This effect was more robust and lasted longer in females. Sham EA Pro had no effect on tumor-induced hyperalgesia. Data shown as mean % response ± SEM, **P < 0.01, and ****P < 0.0001.

While EA treatment given only once at PID 1 showed a trend towards reducing tumor-associated nociception when compared to the tumor-no EA groups, this did not reach statistical significance in either male or female animals at any of the time points tested (Figure 3(a)). Sham EA-Once/1 had no effect on tumor-induced mechanical hyperalgesia (Figure 3(b)).

#### 3.3.5. EA-PRO (Electroacupuncture Administered 3 Times prior to Tumor Cell Implantation)

Lastly, early prophylactic treatment produced a robust antihyperalgesic effect at PID 3, 7, and 10 in females and at PID 3 and 7 in male animals as compared to the tumor-no EA male and female control groups (Figure 3(c); **P < 0.01, ***P < 0.001, ****P < 0.0001). This antihyperalgesic effect was absent at all of the time points tested after PID 10 (PID 14, 17, and 21). Sham EA given prophylactically (Sham EA Pro) had no effect on tumor-induced nociception.

#### 3.3.6. EA Controls

**Figure 4:**
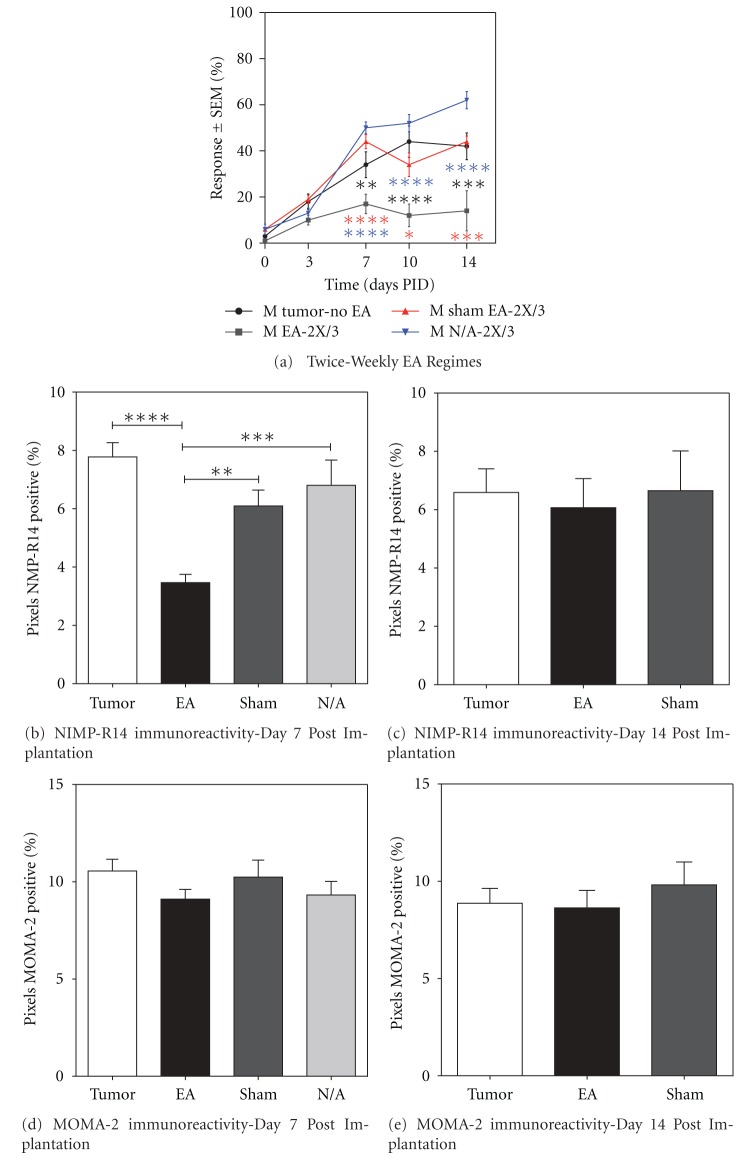
Graphs showing (a) the effect of EA-2X/3, sham EA-2X/3 and twice weekly non-acupoint electroacupuncture (N/A-2X/3) treatment on tumor-induced hyperalgesia in the mice used for this experiment and ((b) to (e)) the effects of EA-2X/3 on neutrophils and macrophages at the osteosarcoma tumor site of male (M) mice at PID 7 and 14 (*n* = 5/group). EA-2X/3, but neither Sham EA-2X/3 nor N/A-2X/3, significantly decreased tumor-induced hyperalgesia at PID 7, 10 and 14 ((a); *n* = 10–15/group). Data in “(a)” shown as mean % response ± SEM; *P < 0.05, ****P < 0.0001 for N/A-2X. Sections were immunostained with NIMP-R14 an antibody specific for neutrophils (b) and (c) or with MOMA-2 for macrophages (d) and (e). The average percent Pixel Density (PD) of neutrophils (b), (c) and macrophages (d), (e) within the tumor were calculated from the 40 *μ*m thick immunostained sections as described in Section 2. Each bar represents the average % PD (mean ± SEM) of neutrophils or macrophages at the tumor site for animals treated with no EA, EA-2X, Sham-EA-2X, or EA applied to a non-acupoint (M N/A-2X). Images were analyzed with ImageJ software (NIH, Bethesda, MD)—see Figure 5. EA-2X/3 treatment produced a significant reduction in the average neutrophil density within the tumor periphery at PID 7 as compared to tumor-no EA (****P < 0.0001), Sham-2X/3 (**P < 0.01), and N/A-2X/3 (***P < 0.001), returning to control densities by PID 14; *P* > 0.05 for all groups. Average densities of macrophages remained comparable in all groups regardless of the time point measured (*P* > 0.05).

Administration of sham EA (Sham EA-1X/7, Sham EA-Once/1, Sham EA-2X/3, Sham EA-2X/5, and Sham EA Pro) had no significant effect on tumor-induced hyperalgesia at any time point tested as indicated previously (Figures 1(c), 2(b), 2(d), 3(b), and 3(d); *P* > 0.05). Since EA-2X/3 produced the most robust and longest lasting analgesic effect, we repeated this regimen in a second set of animals that were used for subsequent immunohistochemical quantification of neutrophil and macrophage density within the tumor (see the following). Moreover, since this anti-hyperalgesic effect was evident by PID 7 in males, but not until day 10 in females, this set of experiments was only performed on male tumor and control mice. The von Frey scores for this group together with those of a sham EA-2X/3 and a non-acupoint treatment group (N/A-2X/3) are shown in Figure 4(a). It is noteworthy that stimulation of a nonacupoint located on the base of the tail failed to produce an analgesic effect at any of the time points tested.

### 3.4. Reduction in Neutrophil but Not Macrophage Density following EA-2X/3 Treatment

**Figure 5:**
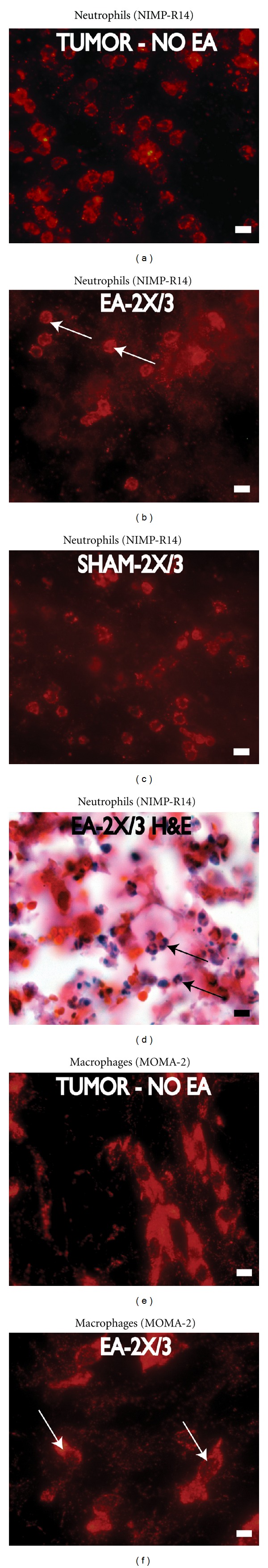
Fluorescent immunohistochemistry and H&E staining for neutrophils (a)–(d) or macrophages (e)-(f) in peripheral tumor hind paw tissue. All images were magnified to 40X with a Nikon 80i fluorescent microscope and captured with ACT-1 software (Nikon, Melville, NY). White arrows (b), (f) indicate labeled immunocytes; black arrows (d) indicate neutrophils stained with H&E. EA-2X/3 significantly reduced the number of neutrophils at the tumor site ((b) compared to (a)), but produced no change in the numbers of macrophages ((e) versus (f)). Scale bars represent 20 *μ*m.

In order to evaluate the effects of EA on tumor-associated inflammation, we utilized immunohistochemistry to quantify the density of neutrophils and macrophages present in sections through the tumor site in nontreated mice compared to those treated with EA-2X/3, sham-EA twice per week (Sham-2X/3), or non-acupoint EA twice per week (N/A-2X/3). EA-2X/3 treatment produced a significant reduction in the average neutrophil density (Figures 4(b) and 5(a)) within the tumor at PID 7 as compared to the tumor-no EA (****P < 0.0001), Sham-2X/3 (**P < 0.01), and N/A-2X (***P < 0.001) groups. Image analysis measurements for average neutrophil density on PID 7 for EA-2X/3, tumor-no EA, Sham-2X/3, and N/A-2X/3 were 3.33 ± 0.32, 8.47 ± 0.63, 6.10 ± 0.55, and 6.80 ± 0.87, respectively (Figure 4(b)). By PID 14, neutrophil densities returned to that observed in the tumor-no EA group (Figure 4(d); *P* > 0.05). Average densities of macrophages remained comparable in all groups regardless of the time point (*P* > 0.05; Figures 4(c), 4(e), and 5(b)). No nonspecific staining was observed in any of the immunohistochemical control slides.

### 3.5. Effect of EA-2X/3 on Tumor-Associated Prostaglandin *E*_2_ (PGE_2_)

**Figure 6:**
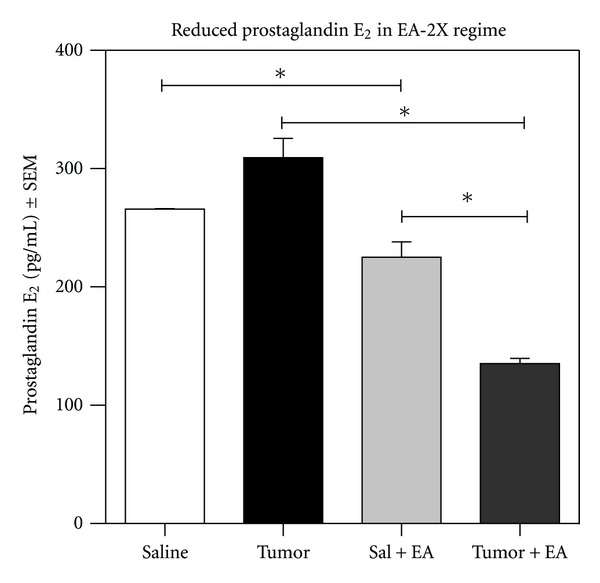
Mean PGE_2_ concentration in pg/mL as measured by ELISA from fibrosarcoma tumor microperfusate samples (*n* = 3/group). Data are expressed as mean ± SEM. Mean tumor PGE_2_ concentration significantly decreased following application of EA-2X beginning at PID 3. In addition, EA reduced the PGE_2_ concentration in saline injected control animals. Mean PGE_2_ concentrations for Saline, Tumor, Sal + EA-2X and Tumor + EA-2X were 265.80 ± 0.20, 309.20 ± 16.33, 225.20 ± 12.80, and 135.20 ± 4.36, respectively (*P < 0.05, Saline + EA-2X versus Tumor + EA-2X; *P < 0.05, Tumor versus Tumor + EA-2X; *P < 0.05, Saline versus Sal + EA-2X).

We determined PGE_2_ concentrations in microperfusates taken from male C3H mice both before and again following 30 minutes of EA stimulation. The data indicate that the concentration of PGE_2_ is increased in the extracellular fluid at the tumor site compared to saline (nontumor cell) injected control animals. Importantly, the mean tumor PGE_2_ concentration significantly decreased following application of EA-2X/3 (Figure 6). Finally, saline injected control animals receiving EA-2X/3 treatment also showed a reduction in PGE_2_ concentration compared to saline injected control animals receiving no EA treatment. Mean PGE_2_ concentrations for Saline, Tumor, Sal + EA-2X, and Tumor + EA-2X were 265.80 ± 0.20, 309.20 ± 16.33, 225.20 ± 12.80, and 135.20 ± 4.36, respectively (*P < 0.05, Saline + EA-2X versus Tumor + EA-2X; *P < 0.05, Tumor versus Tumor + EA-2X; *P < 0.05, Saline versus Sal + EA-2X).

## 4. Discussion

Acupuncture has been shown to be efficacious in the management of the side effects associated with cancer chemotherapy, and radiotherapy and more recently several studies suggest that it can also be used effectively to treat cancer pain itself [5, 7, 35, 46–49]. Yet, despite the number of studies documenting a positive effect of acupuncture on tumor pain [35] two recent systematic reviews of randomized clinical trials have failed to provide strong support for a significant effect of acupuncture on cancer pain [50, 51]. These reviews do not speak as much to the ineffectiveness of acupuncture as they do to the inadequacies and lack of rigor in the majority of acupuncture-associated randomized clinical trials in the literature, which suffer from methodological flaws. These flaws include inadequate study design, use of improper controls, poor reporting of results, lack of or inadequate blinding, small sample size, and overestimation of the results. Thus, the problem of establishing EA's effectiveness in treating cancer pain or other types of pain may not lie within the EA approach itself, but rather within setting proper methodological parameters, creating the appropriate study design and sample size and establishing proper controls [5]. In this regard, there are several advantages to studying the potential antinociceptive effects of acupuncture in rodent models of cancer pain, because it is possible to control tumor development, sample size, methodological parameters, and the study design in a lab environment.

 The majority of previous studies that have used animal models to analyze the potential anti-nociceptive effects of acupuncture on tumor-induced nociception provide strong support for the hypothesis that acupuncture can relieve tumor-induced pain [6, 51–54]. Using an osteosarcoma bone cancer model, we demonstrate that different EA regimens all produce an immediate, but temporary effect lasting for approximately 30min after treatment. This is consistent with the findings of Mao-Ying et al. [53] who demonstrated that a single EA treatment on day 8 after implantation of melanoma cells into the hind paw produced a significant analgesic effect immediately after the treatment reaching a maximum effect within 15 to 20 min after EA. With respect to long-term effects, we show that only EA given twice weekly beginning at PID 3 or prophylactically prior to tumor cell implantation significantly reduced tumor-induced mechanical hyperalgesia over days to weeks. Thus, one of the novel outcomes of the present study is that different treatment regimens produce different long-term anti-nociceptive results and that early treatment given twice a week produces the most long-lasting analgesia particularly in females. Mao-Ying et al. [53] also showed that repeated EA treatments (given once every other day starting at PID 8 after melanoma implantation) showed a persistent analgesic effect, while it showed no therapeutic effect when starting from day 16. This lack of effect when EA treatment is started later is reminiscent of our finding that twice weekly treatment starting at PID 5, rather than PID 3 negates the long-term analgesic effect. The difference in timing between the two studies (treatment beginning at PID 3, but not PID 5 is effective for osteosarcoma versus PID 8, but not PID 16 for melanoma) probably reflects a combination of the different tumor models used (bone cancer versus cutaneous melanoma) and the different behavioral tests employed (von Frey versus radiant heat). In a study by Lee and coworkers [52], EA was administered to ST-36 daily for 9 days after tumor cell injection, and they reported that EA treatment significantly prolonged paw withdrawal latency from 5 days following inoculation. Similarly Zhang et al. [54] administered EA daily at GB-30 between PID 14 and 18 in a rat prostate bone cancer model and saw a significant inhibition of cancer-induced thermal hyperalgesia. While we did not attempt daily treatment in the present study, our results support the concept that early and frequent treatment (in our case EA given twice a week beginning on day 3 subsequent to tumor cell implantation) has long-lasting analgesic effects with respect to cancer pain. While we cannot make solid recommendations for the treatment of human cancer pain based on the present results in mice, our data would suggest that the earlier treatment is begun, the more effective the analgesic effect will be. Moreover, an acupuncture treatment regime that is given twice per week may be more effective than one given once per week or less often. Although we tested the effect of EA-2X/3 treatment on inflammation and PGE_2_ release at the tumor site, we did not test the effect of other treatment regimes on these parameters, which probably contribute to EA-2X/3′s analgesic effect. Clearly more work needs to be done to determine if other treatment regimes also effect tumor-associated inflammation and/or prostaglandin concentrations.

With the exception of the work by Lund and Lundberg [55] there are no controlled studies in the literature that have actually examined the differences in the effects of acupuncture on pain in males versus females. Lund and Lundberg [55] hypothesized that gender contributes to the variable results of pain alleviation in response to acupuncture, and they showed that sensory thresholds are changed in men, but not women following acupuncture treatment. Our results show that the anti-nociceptive effect of both prophylactic EA and EA-2X/3 treatment lasts longer in female animals compared to males, but the analgesic effect of EA-2X/3 treatment begins early and is more robust in male animals. In contrast both EA-1X/7 and EA-Once/1 treatments had little effect on tumor-induced pain with only female animals showing a significant decrease at PID 7 following EA-1X/7 treatment. Collectively, this data suggests that sex differences do exist with respect to EA treatment and are consistent with a recent report showing differences in brain activity between men and women during acupuncture treatment [56]. While our data is not directly prescriptive for human acupuncture treatment regimes, it would suggest that early treatment of female cancer patients may be more beneficial, while males may benefit from more frequent treatments. However, until further study of EA sex differences in human patients is performed, it would be prudent to consider the factors of gender and sex when planning acupuncture treatment, as the response to acupuncture treatment may differ between men and women. Nonetheless, it is critical that future clinical acupuncture trials, those that are well blinded and include placebo acupuncture, take into account potential sex difference.

Over the past decade evidence has accumulated indicating that there is a strong link between inflammation and cancer [17, 22–24]. Infection-driven inflammation has been implicated in the pathogenesis of ~15%–20% of human tumors [22], chronic inflammation appears to proceed tumorigenesis for many types of tumors [25, 57], most tumors appear to have an inflammatory component, and increasing evidence indicates that leukocyte infiltration can promote tumor phenotypes, such as angiogenesis, growth, and invasion [58]. Moreover, a wide array of proinflammatory cytokines, chemokines, prostaglandins and extracellular proteins are closely involved in the premalignant and malignant conversion of cells on a background of chronic inflammation [22]. While the interaction of these inflammatory mediators and effector cells with carcinogenesis and tumor progression is complicated, it is likely that the release of these mediators contributes to tumor pain [12, 59]. 

Acupuncture stimulation has been previously shown to reduce inflammation [60–62] and also to reduce inflammatory-induced pain [63]. The ST-36 acupoint has been used in many studies that have evaluated the antinociceptive effects of acupuncture, and this same acupoint has also been used to investigate the potential anti-inflammatory capabilities of acupuncture [61, 64]. Electroacupuncture at ST-36 decreases COX-2 expression in models of pain and inflammation [65]. In the present study, we found that EA administered at ST-36 for 30 min twice per week significantly reduced the density of neutrophils at the tumor site and that this correlated with EA's anti-nociceptive effects. While we did not determine the mechanisms by which EA reduced neutrophil density in the context of the present study, previous work from our laboratories has shown that EA can reduce neutrophil migration into sites of inflammation via activation of sympathetic pathways, release of noradrenaline, and activation of beta adrenergic receptors on neutrophils to inhibit their migration [60, 66]. Similarly, da Silva and colleagues [67] showed that stimulation of the SP-6 acupoint inhibited neutrophil infiltration in a mouse peritonitis model. While this is a likely mechanism underlying the EA-induced reduction in neutrophil densities at the tumor site, it is also possible that neutrophil density was reduced by apoptosis, since reduction through apoptotic mechanisms plays an important role in maintaining homeostasis in neutrophil numbers, which are short-lived cells and readily go into apoptosis [68]. 

 While a reduction in neutrophil density at the tumor site may be partially responsible for the EA-associated reduction in tumor-induced nociception, this is by no means the entire story. As indicated in Figure 5(b), the density of neutrophils at the tumor site returned to that observed in tumor control animals with no EA by 14 days after tumor cell implantation, a time point at which there is still a significant EA-induced anti-nociceptive effect on tumor-induced mechanical hyperalgesia. Thus, other anti-nociceptive mechanisms are at play at the 14-day postimplantation time point that apparently does not involve a reduction in neutrophil density. Another potential candidate for tumor-induced nociception is prostaglandin E_2_ (PGE_2_). When tissue damage or inflammatory conditions are present, prostaglandins are synthesized and released in response. We have shown in the present study that the concentration of PGE_2_ decreases in tumor microperfusates by 30 min following EA-2X, and it is possible that this may be one of the factors that contribute to the reduction in tumor-induced nociception. Since PGE_2_ has been shown to cause pain following intraplantar injection in mice [69], it is likely that the tumor-associated increase in PGE_2_ contributes to tumor-induced nociception. The EA-induced reduction in extracellular PGE_2_ concentration at the tumor site may be partially responsible for the anti-nociceptive effect of EA.

## 5. Conclusion

This study examined the effects of five different electroacupuncture (EA) treatment schedules on murine bone tumor-induced hyperalgesia in male and female animals. We show that both early and frequent (EA-2X/3) administration of EA is effective in controlling tumor-induced hyperalgesia, while treatments applied later and less frequently are not. Importantly, we demonstrate that there are sex differences associated with different treatment regimens, which may be important to consider in treating patients clinically. In this regard the analgesic effect of both prophylactic EA and EA-2X/3 treatment lasted longer in female animals, while males showed an earlier and more robust response to EA-2X/3 treatment. Finally, we provide new evidence that EA may decrease tumor-induced pain in part by reducing both tumor-associated inflammation and PGE_2_ production at the tumor site. It is critical that EA treatment schedules be carefully considered in treating acute and chronic pain conditions particularly cancer pain and that potential sex differences be taken into account. Clearly further research needs to be done to better understand bone cancer mechanisms, how inflammation affects bone cancer progression, and if alternative medicine treatment approaches can be found that both relieve cancer pain and reduce tumor burden. 

## Figures and Tables

**Table 1:** Summary of the total number of male and female animals used for each experiment.

Assay	Treatment type	Male (*n*)	Female (*n*)
	Tumor	24	15
Mechanical Hyperalgesia von Frey	Saline	11	7
	EA	52 (10-11/group)	45 (9/group)
	Sham	30 (6/group)	37 (7-8/group)

Immunohistochemistry	Tumor	10	0
	EA	10	0
	Sham	10	0
	N/A	10	0

Microperfusion/ELISA	Tumor	3	0
	Saline	3	0
	Sal + EA	3	0
	Tum + EA	3	0

		186	104

## Glossary

PID: Postimplantationday
EA: Electroacupuncture
PGE_2_: Prostaglandin E_2_
CAM: Complementaryandalternativemedicine.
